# Geographical variation in determinants of high-risk fertility behavior among reproductive age women in Ethiopia using the 2016 demographic and health survey: a geographically weighted regression analysis

**DOI:** 10.1186/s13690-020-00456-5

**Published:** 2020-08-18

**Authors:** Zemenu Tadesse Tessema, Melkalem Mamuye Azanaw, Yeaynmarnesh Asmare Bukayaw, Kassahun Alemu Gelaye

**Affiliations:** 1 Department of Epidemiology and Biostatistics, Institute of Public Health, College of Medicine and Health Sciences, University of Gondar, Gondar, Ethiopia; 2 Department of Social and Public Health, College of Health Sciences, Debre Tabor University, Debre Tabor, Ethiopia

**Keywords:** High-risk fertility behavior, Spatial distribution, GWR, Ethiopia

## Abstract

**Background:**

Maternal and child mortalities are the main public health problems worldwide and both are the major health concern in developing countries such as Africa and Asia. The fertility behavior of women characterized by maternal age, birth spacing, and order, impacts the health of women and children. The aim of this study was to assess the geographically variation in risk factors of high-risk fertility behavior (HRFB) among reproductive-age women in Ethiopia using the 2016 Demographic and Health Survey.

**Methods:**

A total of 11,022 reproductive-age women were included in this study. The data were cleaned and weighted by STATA 14.1 software. Bernoulli based spatial scan statistics was used to identify the presence of pure high-risk fertility behavior spatial clusters using Kulldorff’s SaTScan version 9.6 software. ArcGIS 10.7 was used to visualize the spatial distribution of high-risk fertility behavior. Geographically weighted regression analysis was employed by multiscale geographical using Multiscale geographical weighted regression version 2.0 software. A *p*-value of less than 0.05 was used to declare statistically significant predictors (at a local level).

**Results:**

Overall, 76% with 95% confidence interval of 75.60 to 77.20 of reproductive age women were faced with high-risk fertility problems in Ethiopia. High-risk fertility behavior was highly clustered in the Somali and Afar regions of Ethiopia. SaTScan identified 385 primary spatial clusters (RR = 1.13, *P* <  0.001) located at Somali, Afar, and some parts of Oromia Regional Stateregional state of Ethiopia. Women who are living in primary clusters were 13% more likely venerable to high-risk fertility behavior than outside the cluster. In geographically weighted regression, not using contraceptives and home delivery were statistically significant vary risk factors affecting high- risk fertility behavior spatially. No contraceptive use and home delivery were statistically significant predictors (at the local level) in different regions of Ethiopia.

**Conclusion:**

In Ethiopia, HRFB varies across regions. Statistically, a significant-high hot spot high-risk fertility behavior was identified at Somali and Afar. No contraceptive use and home delivery were statistically significant predictors (at a local level) in different regions of Ethiopia. Therefore, policymakers and health planners better to design an effective intervention program at Somali, and Afar to reduce high-risk fertility behavior and Special attention needs about health education on the advantage of contraceptive utilization and health facility delivery to reduce high-risk fertility behavior.

## Background

Maternal and child mortalities are the main public health problems worldwide, and both are the major health concern in developing countries such as Africa and Asia [1]. Globally, 830 women die from preventable causes related to pregnancy and childbirth (every year) of which 99% of all deaths occur in developing countries [2]. Ethiopia is one of the countries hosting the highest maternal mortality ratio with 412 deaths per 100,000 live births every year according to 2016 Ethiopian Demographic and Health Surveys (EDHS) reports and most of the deaths were attributed to high-risk fertility behavior [3]. Under the sustainable development goal (SDG), maternal mortality has been targeted to be below 70 deaths per 100,000 live births at the end of 2030 [4].

The global population is rapidly increasing and according to the 2016 report, the total fertility rate was 2.5 and 4.8 per woman globally and in Ethiopia, respectively [5]. The fertility behavior of women characterized by maternal age, birth spacing, and order impacts the health of women and children [6, 7]. High-risk fertility behavior associates with numerous unfavorable child and maternal health outcomes such as chronic undernutrition, anemia, and child mortality [8–10]. Different studies showed that high-risk fertility behaviors associate with chronic undernutrition and anemia among under-five children. Besides, these behaviors are related to adverse birth outcomes such as stillbirth, low birth weight, and prematurity [9, 11–13]. Whenever the birth interval got narrower (less than 24 months), the chance of child morality increased sharply as compared to long spaced birth intervals [14]. The risk of infant mortality from teenage mothers increased by 30% as compared to those who gave birth between the ages of 20 and 30. The problem is higher in developing countries where healthcare services are inaccessible, high unmet family planning needs, and low socio-economic conditions [8, 11–13, 15, 16]. In addition, early marriage is another problem for high-risk fertility behavior in Ethiopia and other low and middle-income countries [15].

Different factors are associated with high-risk fertility behavior, such as socio-demographic characteristics (residence, religion, education level, and marital status). Reproductive health characteristics such as a history of child death, facility delivery, and family panning utilizations are factors associated with high-risk fertility behavior [10–13, 15–18]. The government and stakeholders made tremendous efforts such as increasing health services accessibility and coverage, providing exempted maternal health services, and postnatal care follow-up [19]. Although different studies have been conducted to assess the magnitude and effects of high-risk fertility behaviors, no national study has accounted for geographical variability risk factors of high-risk fertility behavior.

As to the investigator’s knowledge, this first geographically weighted analysis on high-risk fertility behavior and geographically vary risk factors among reproductive-age women in Ethiopia have been provided. This study could help healthcare planners and policymakers to design evidence-based interventions and appropriate allocation of resources in hot spot areas.

## Methods

### Study design, area, and period

A population-based cross-sectional study was conducted using EDHS 2016. Ethiopia is situated in the Horn of Africa. It has 9 Regional states (Afar, Amhara, Benishangul-Gumuz, Gambela, Harari, Oromia, Somali, Southern Nations, Nationalities, and People’s Region (SNNP) and Tigray) and two city Administrative (Addis Ababa and Dire-Dawa). The survey was conducted from January 18, 2016, to June 27, 2016, using a countrywide representative sample that provides estimates at the national and regional levels and urban and rural areas.

### Sources and study populations

The source population was all reproductive age group women, 5 years preceding the survey.

A total of 15,683 women aged 15–49 years were interviewed and 11,023 women included in the analysis. In the 2016 EDHS, a total of 645 clusters (EAs) (202 urban and 443 rural) were selected with a probability proportional to each EAs size and independent selection in each sampling stratum (urban = 1215 and rural =9807). Among a total selected cluster that coordinate clusters in which unable to obtain organized data and having missing data were excluded for the analysis. Finally, a total of (185 urban and 413 rural) clusters were used for this study. Among the selected clusters, 11,023 (urban = 1215 and rural =9807) weighted women were included in this study. The recorded data were accessed at www.measuredhs.com on request with the help of ICF International, Inc.

### Data collection tools and procedures

Ethiopian Demographic and Health Survey data were collected by two-stage stratified sampling. Each region of the country was stratified into urban and rural areas, yielding 21 sampling strata. In the first stage, 645 EAs were selected with probability proportional allocation to enumeration area size with independent selection in each sampling stratum. In the second stage of selection, a fixed number of 28 households per cluster were selected through, systematic sampling technique from the newly created household listing. The detailed sampling procedure is available in the Ethiopian Demographic and Health Survey reports from Measure DHS website (www.dhsprogram.com).

### Outcome variable

For this study, three parameters were considered (maternal age at the time of delivery, birth order, and birth interval), to define the high-risk fertility behaviors. Three exposure variables were defined for this analysis. Any high-risk fertility behavior versus non-risk coded as 1/0, respectively. The presence of any of the following four conditions was termed high-risk fertility behavior:

1. Mothers aged less than 18 years at the time of delivery
2. Mothers aged over 34 years at the time of delivery
3. The latest child born less than 24 months after the previous birth
4. Latest child of order three or higher

The definition of ‘high-risk fertility behaviors’ adopted by the 2016 EDHS was applied [3]. The dependent variable in this analysis was high-risk fertility behavior (proportion in the cluster).

### Predictor variables

From the 2016 EDHS datasets, independent variables such as the proportion of rural, male sex, religion, education, occupation, anemia, wealth index, ANC visit, home delivery, media exposure, and wanted pregnancy were taken as independent variables.

### Data management and analysis

The data were cleaned by STATA version 14.1 software and Microsoft excel. Sample weighting was done for further analysis.

### Spatial autocorrelation and hot spot analysis

Spatial autocorrelation (Global Moran’s I) statistic measure was used to assess whether HRFB among reproductive-age women was dispersed, clustered, or randomly distributed in Ethiopia. Moran’s I values close to − 1 indicated the low proportion of HRFB and dispersed, close to + 1 indicates clustered, and if Moran’s I value zero indicates randomly distributed [20]. A statistically significant Moran’s I value (*p* <  0.05) had a chance to reject the null hypothesis, indicating the presence of spatial autocorrelation. Hot Spot Analysis (the Getis-Ord Gi* statistic) of the z-scores and significant *p*-values tells the features with either hot spot or cold spot values for the clusters spatially. we used high-high clusters to investigate the local level cluster locations of HRFB.

### Spatial interpolation

The spatial interpolation technique is used to predict HRFB proportion among reproductive-age women for unsampled areas in the country based on sampled EAs. For the prediction of unsampled EAs, we used deterministic and geostatistical Empirical Bayesian Kriging spatial interpolation techniques. Ordinal Kriging method of Gaussian distribution was used [21].

### Spatial scan statistics

Bernoulli based model spatial scan statistics was employed to determine the geographical locations of statistically significant clusters for HRFB using Kuldorff’s SaTScan version 9.6 software [22]. The scanning window that moves across the study area in which HRFB was taken as cases and no HRFB were taken as controls to fit the Bernoulli model. The default maximum spatial cluster size of < 50% of the population was used as an upper limit, allowing both small and large clusters to be detected and ignored clusters that contained more than the maximum limit with the window’s circular shape. Most likely, clusters were identified using *p*-values and likelihood ratio tests based on the 999 Monte Carlo replications.

### Geographically weighted regression analysis

Ordinary Least Square regression (OLS) model is a global model that estimates only one single coefficient per explanatory variable over the entire study area. Global models assume factors that affect HRFB were geographically stationary. The assumption of geographic independence relaxes by geographically weighted regression analysis. A geographically weighted regression model is an extension of the OLS regression model. It gives local parameter estimates to reflect changes over space in the association between an outcome and explanatory variables [23].

For the interest of geographically weighted regression analysis, the aggregated proportion of HRFB among reproductive-age women and all the predictor variables were calculated for each cluster. To determine the predictor variables for HRFB among reproductive-age women, we used a geographically weighted regression model.

To check the spatial dependency assumption, the explanatory analysis was performed first by Arc GIS 10.7 software. Statistically significant (*P* <  0.01) Koenker (BP) statistic indicates that the relationships modeled are not consistent (either due to non-stationarity or heteroskedasticity). Multicollinearity (Variance Inflation Factor < 7.5) was checked to exclude redundancy among explanatory variables. In spatial dependency, the coefficient of the predictor variable varies locally; the predictor variables may or may not significant locally. The model structure of geographically weighted regression written as,

$$ Yi=\beta 0\left( ui, vi\right)+\sum \mathrm{k}\upbeta \mathrm{k}\left(\mathrm{ui},\mathrm{vi}\right)\mathrm{Xik}+\mathrm{i} $$

Where Y_i_ is the response variable, (u_i_, v_i_) denotes the coordinates of the i^th^ point in space, β_0_ is the intercept at the (u_i_, v_i_) coordinate, β_k_ is the coefficient of the covariate X at the (u_i_, v_i_) coordinate, and_i_ is the random error term.

### Calibration of the model

Multiscale Geographically Weighted Regression (MGWR) version 2.0 software was used to calibrate the parameter estimates of the Geographically Weighted Regression model [24]. The new version of GWR is termed Multiscale Geographically Weighted Regression (MGWR), potentially providing a more flexible and scalable framework for examining multiscale processes. Adaptive bi-square kernels were used for geographical weighting to estimate local parameter estimates. The ‘golden section search ‘method was used to determine the best bandwidth size based on corrected Akaike’s Information Criterion (AICc), and the bandwidth with the lowest AICc was used to determine the best fit model for local parameter estimates.

Geographical variability for each coefficient can be assessed by comparing the AICc between the GWR model and the global OLS regression model. The corrected Akaike’s Information Criterion (AICc) was obtained by minimizing the Akaike Information Criteria (AIC) which is;

$\mathrm{AICc}=2{\mathrm{nlog}}_{\mathrm{e}}\left(\upsigma \hat{\mkern6mu} \right)+{\mathrm{nlog}}_{\mathrm{e}}\left(2\uppi \right)+\left\{\frac{\left(\mathrm{n}+\mathrm{tr}\left(\mathrm{s}\right)\right)}{\left(\mathrm{n}\hbox{-} 2\hbox{-} \mathrm{tr}\left(\mathrm{s}\right)\right)}\right\}\dots \dots \dots \dots$ [23]where n is the sample size, σˆ is the estimated standard deviation of the error term, and tr(**S**) denotes the trace of the hat matrix, which is a bandwidth function. Finally, local parameter estimates were plotted on Arc GIS 10.7(ESRI Inc., Redlands, CA, USA, version 10.7) software.

## Results

### Prevalence of high-risk fertility behavior

A total of 11,022 women were included, with 643 of clusters nested in 11 regions. This study revealed that the magnitude of HRFB among women was 76.3% with 95% CI: (75.6, 77.2). The prevalence of HRFB among an urban and rural place of residence of women was 66.51 and 77.59%, respectively (Table 1).

**Table 1:** Socio-economic and demographic variables considered for the global regression model of High-risk fertility behavior in Ethiopia using Ethiopian Demographic and Health Survey, 2016

Variables	Description	Proportion
Place of residence	Proportions of women with Rural place of residence	0.89
Sex	Proportions of males	0.52
Religion	Proportions of women of Orthodox Christian religion followers	0.34
	Proportions of women of Muslim religion followers	0.41
	Proportions of women of protestant religion followers	0.21
Educational status of women	Proportions of women with no Education	0.66
Occupation of women	Proportions of women with no work	0.56
Anemia status	Proportions of women with Anemia	0.30
Wealth status	Proportions of women with low economic status	0.46
Antenatal follow up	Proportions of women with no ANC follow up	0.25
Place of delivery	Proportions of women with home delivery	0.72
Media exposure status	Proportions of women with no media exposure	0.09
Wanted pregnancy	Proportions of not wanted pregnancy	0.25

### Spatial autocorrelation of high-risk fertility behavior in Ethiopia

**Fig. 1:**
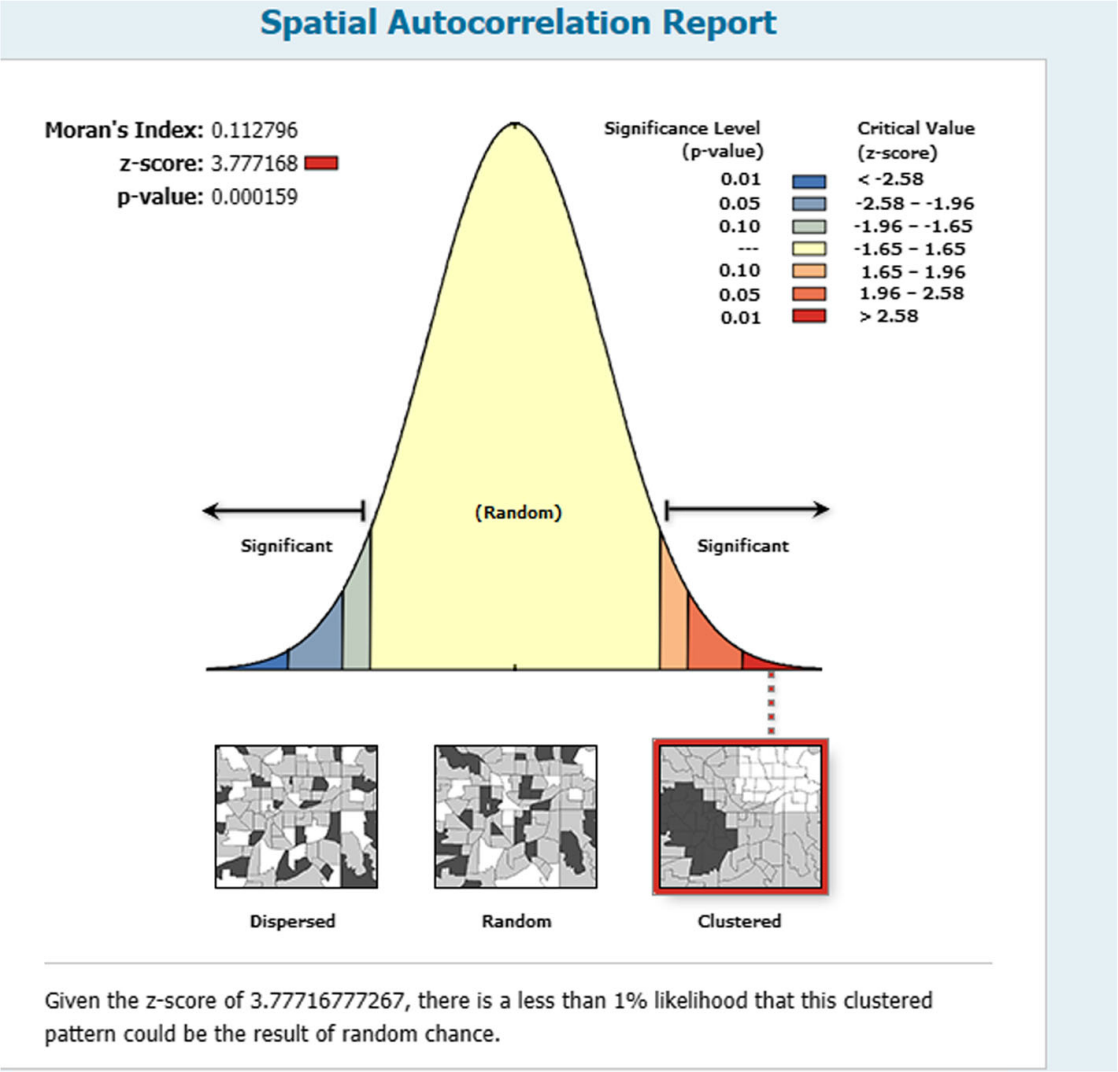
Spatial autocorrelation of elevated risk fertility behavior in Ethiopia, 2016

This study revealed that the spatial distribution of HRFB was found to be non-random in Ethiopia with Global Moran’s I 0.113 (p <  0.001) (Fig. 1).

The cluster patterns (on the right sides) show high rates of HRFB occurrence over the study area. The outputs have been automatically generated keys on the right and left sides of each panel. Given the z-score of 3.78 indicated that there is less than 1% likelihood that this clustered pattern could result from random chance. The bright red and blue colors to the end tails indicate an increased significance level. The table shows that the observed value is greater than the expected value and *P*-value is < 0.05, it is statistically significant.

### Incremental spatial l autocorrelation among reproductive-age women in Ethiopia

**Fig. 2:**
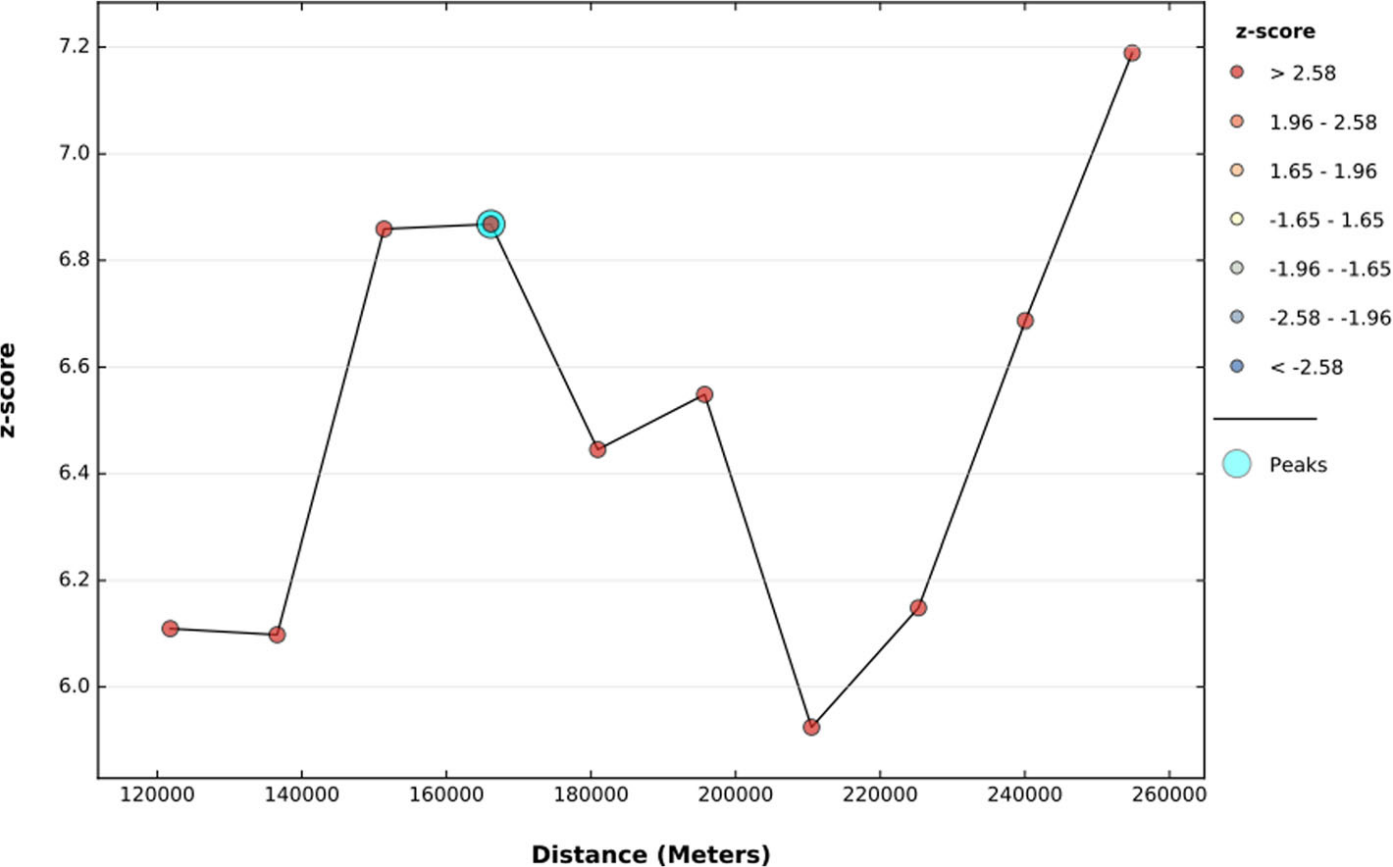
The spatial autocorrelation of high-risk fertility behavior among reproductive age group women in Ethiopia by a function of distance using Ethiopian Demographic and Health Surveys 2016

To determine spatial clustering for HRFB, global spatial statistics were estimated using Moran’s I value. As shown in the figure below a statistically significant z-scores indicated at 166 Km distances where spatial processes promoting clustering are most pronounced. The incremental spatial autocorrelation demonstrates that 10 distance bands were detected with a beginning distance of 121,813 m. The spatial distribution of HRFB among reproductive-age women in Ethiopia was found non-random with a Global Maran’s I was 0.11 and *p*-value 0.0001. The z-score of 3.77 shows a less than 1% likelihood that this high-clustered pattern could be the result of random chance. (Fig. 2).

### Hot spot (Getis-Ord Gi) analysis

**Fig. 3:**
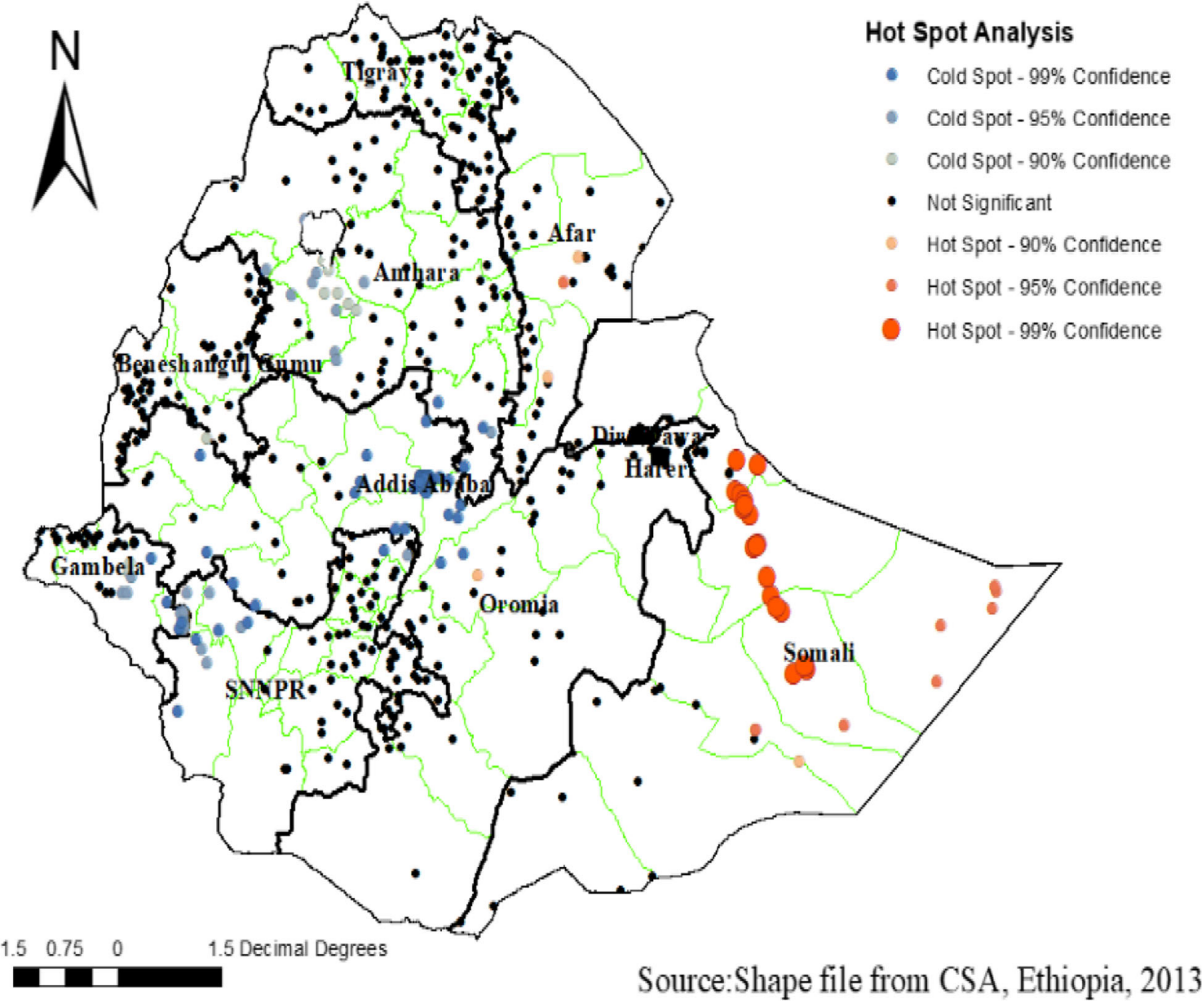
Hot spot analysis of high-risk fertility behavior among women within 5 years preceding the survey in Ethiopia, using Ethiopian Demographic and Health Surveys 2016

As shown in the figure below, the red color indicates the more intense clustering of high (hot spot) proportion HRFB preceding the survey period. A high proportion of HRFB was clustered at the Somali and Afar region of Ethiopia. Whereas, Amhara, SNNPR, and Addis Ababa regions of Ethiopia were less risk area. (Fig. 3).

### Spatial sat scan analysis of high-risk fertility behavior among women across regions of Ethiopia, 2016

**Fig. 4:**
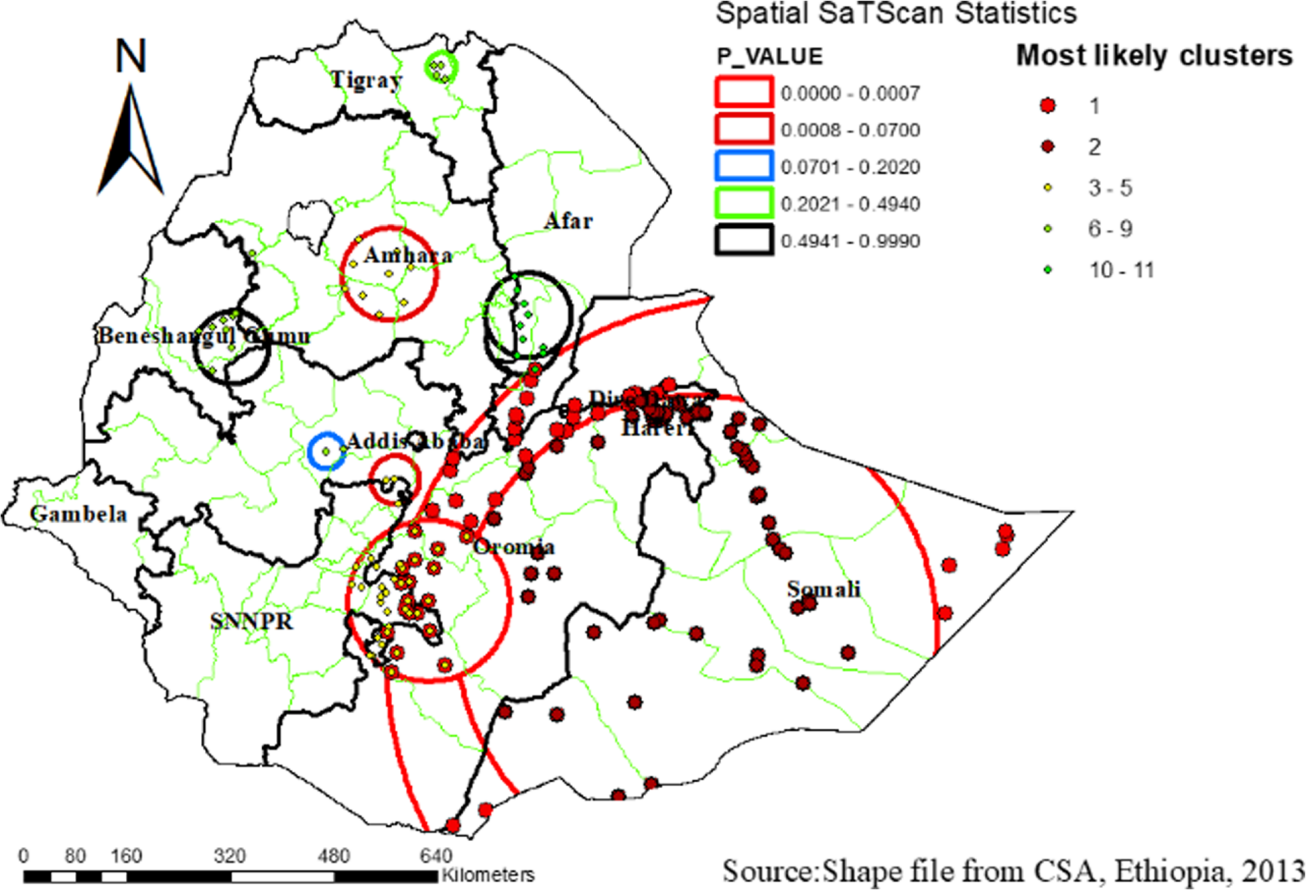
Spatial Sat Scan analysis of high-risk fertility behavior among women across regions of Ethiopia, using Ethiopian Demographic and Health Surveys 2016

Most likely primary clusters and secondary clusters of HRFB were identified. A total of 383 significant clusters were identified. Of these, 181 of them were most likely primary clusters and 102 were secondary clusters. The primary clusters’ spatial window was located in the Somali, Eastern Oromia, Dire Dawa, and Harari region which was centered at 5.848373 N, 43.527981 E with 569.73 km radius, and Log-Likelihood ratio (LLR) of 65.24, at *p* < 0.001. It showed that women within the spatial window had 1.13 times higher risk of HRFB than women outside the window. The secondary clusters’ spatial window was typically located in the central part of the Amhara region. Which was centered at 11.287790 N, 38.406887 E with 71.42 km radius, and LLR of 9.46 at *p*-value 0.032? It showed that women within the spatial window had a 1.16 times higher risk of HRFB than women outside the window (Fig. 4 and Table 2).

**Table 2:** Primary and Secondary SaTScan analysis result of high-risk fertility behavior among reproductive-age women in Ethiopia Ethiopian Demographic and Health survey 2016

Cluster type	Significant Enumeration Areas (clusters) detected	Coordinates/Radius	Populations	Cases	RR	LLR	***P-***value
Primary	164, 358, 85, 138, 278, 492, 92, 543, 490, 146, 318, 187, 171, 198, 95, 556, 497, 520, 480, 521, 588, 553, 458, 208, 77, 214, 251, 394,573, 239, 116, 629, 22, 286, 568, 277, 289, 33, 472, 452, 527, 377,64, 439, 186, 57, 8, 210, 454, 513, 436, 501, 68, 212, 580, 483, 133,587, 115, 500, 240, 194, 418, 58, 29, 622, 321, 44, 179, 534, 607, 257, 387, 56, 397, 157, 228, 28, 614, 393, 60, 396, 443, 173, 357, 566, 238, 419, 269, 383, 329, 495, 288, 381, 610, 372, 1, 473, 453, 378, 630, 242, 523, 281, 166, 642, 311, 557, 441, 30, 594, 202, 613,74, 380, 519, 352, 273, 535, 471, 631, 151, 307, 5, 111, 185, 282,444, 514, 606, 390, 27, 493, 385, 224, 467, 43, 476, 644, 363, 190, 546, 93, 101, 140, 25, 529, 123, 412, 245, 7, 506, 319, 333, 422, 122, 562, 491, 213, 34, 71, 518, 26, 49, 619, 524, 405, 51, 82, 230, 468, 564, 576, 313, 365, 589, 438, 316, 149, 39, 12, 398, 125, 391,522, 600, 336	(5.848373 N, 43.527981 E) / 569.73 km	3450	2870	1.13	64.24	< 0.001
Secondary	278, 318, 187, 358, 85, 164, 556, 480, 492, 543, 138, 490, 92, 198, 171, 95, 497, 521, 588, 146, 553, 286, 458, 520, 394, 289, 472, 214, 452, 251, 208, 573, 239, 116, 22, 568, 377, 277, 372, 454, 513, 186, 527, 33, 68, 501, 580, 436, 64, 133, 115, 212, 483, 8, 500, 587, 240, 29, 418, 58, 439, 607, 179, 194, 44, 321, 534, 257, 56, 397, 210, 157, 228, 387, 28, 614, 57, 393, 60, 396, 173, 443, 622, 238, 383, 357, 329, 419, 495, 381, 288, 610, 453, 473, 566, 123, 529, 1, 476, 245, 242, 166	(6.273056 N, 42.688145 E) / 370.11 km	1684	1457	1.16	61.29	< 0.001

### Interpolation of high-risk fertility behavior

**Fig. 5:**
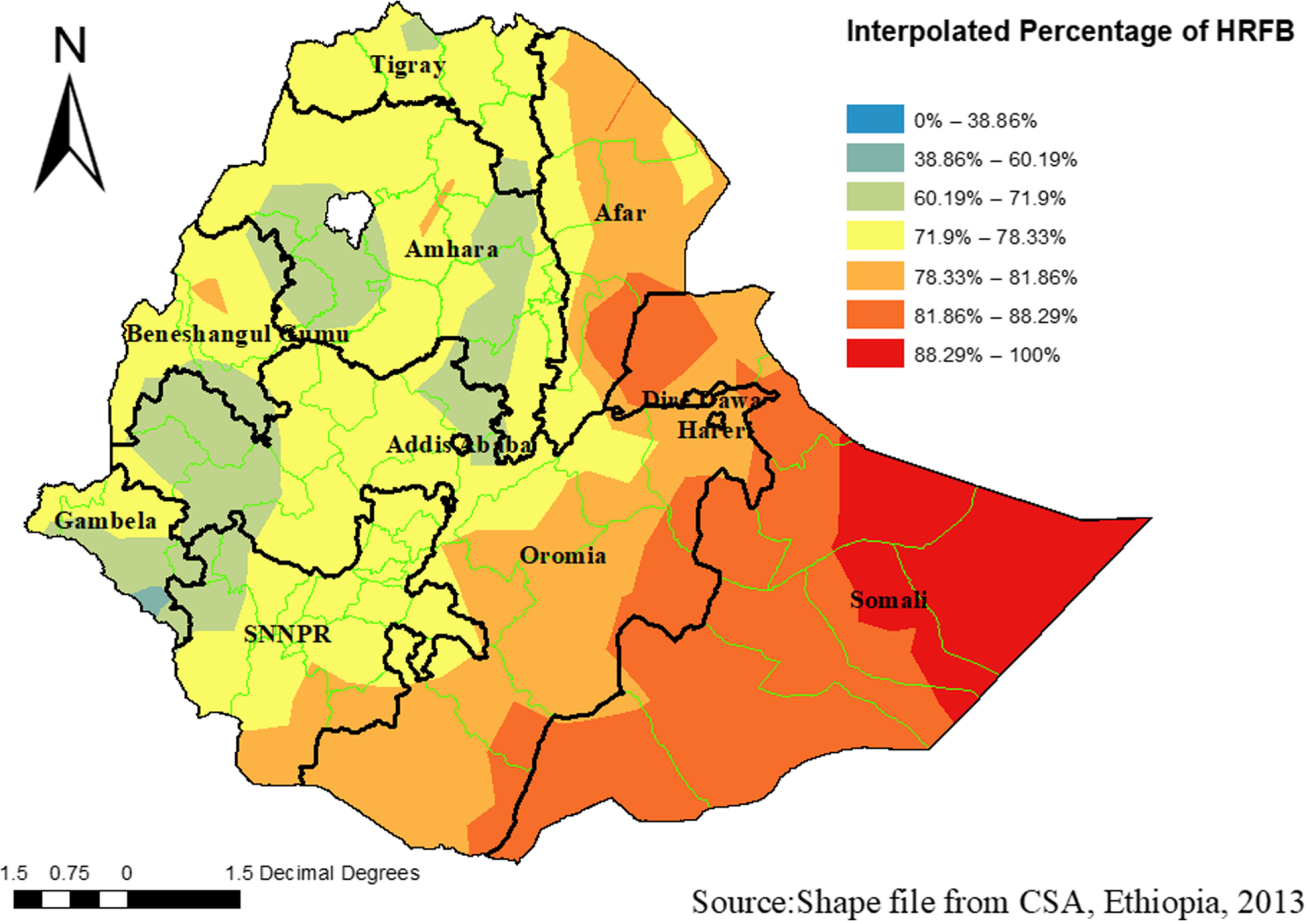
Interpolation of high-risk fertility behavior among reproductive-age women in Ethiopia, Using Ethiopian Demographic and Health Surveys 2016

The predicted high-risk fertility behavior over the area increases from green to red-colored areas. The red color indicates high-risk areas of predicted HRFB, and the green color indicates the predicted low-risk fertility behavior areas. The Somali region, the Afar region, Eastern parts of the Oromia region and center parts of the Benishangul Region were predicted as more risky than other regions. Continuous images produced by interpolating (Kriging interpolation method) HRFB among women. The red color indicates that the predicted high-risk areas and green color show fewer risk areas of HRFB (Fig. 5).

### Geographically weighted regression analysis

Selected predictor variables fitted in the geographically weighted regression model. For model compression, both Ordinary Least Square (OLS) model and Geographical Weighted Regression (GWR) model was fitted. The bandwidth corrected Akakian Information Criteria (AICc) and loglikelihood was considered. Comparing the global model, the GWR model best fits the model with AICc of 1652 compared with 1655 least AICc best approach. As well, the GWR model best explained by the predictor variables log-likelihood also supports it. (Table 3).

**Table 3:** The Model comparison between the OLS model with the GWR model after model fitted in Ethiopia using EDHS 2016

Variable	GLR	GWR
AICc	1655	1652
Residual sum of squares	510.9	502.5
Log likelihood	− 821.4	− 816.3

NB: *AICc* Corrected Akakian Information Criteria.

### Ordinary Least Square (OLS) model result

We found two spatially vary risk factors that affect HRFB among reproductive age group women in Ethiopia from the OLS model. The Global beta coefficients for the proportion of home delivery and no (contraceptive) use were statistically (home delivery beta coefficient = 0.08 *p*-value< 0.001, no (contraceptives) use beta coefficient 0.10 *p-*value < 0.001). When the Koeker test is statistically significant, it indicates relationships between some or all of your explanatory variables and your dependent variable is non–stationary (Koenker (BP)Statistics = 47.8 *p-*vale < 0.001). Breusch–Pagan statistic is used to test for heteroskedasticity in a linear regression model (75.5; *p-*value< 0.001) since the test statistic has a *p-*value below an appropriate threshold (*p-*value < 0.05) then the null hypothesis of homoskedasticity is rejected and heteroskedasticity assumed (Table 4).

**Table 4:** Global beta coefficients of the geographic weighted regression model summary results for best non-spatial linear regression model for the proportion of high-risk fertility behavior among reproductive-age women in Ethiopia, 2016

Variable	Coefficient	Std error	***P-***value	Robust Std error	Robust ***P-***value	VIF
Intercept	0.60	0.014	0.000000*	0.017	0.000000*	… ..
Home delivery	0.08	0.03	0.0006*	0.026	0.001*	2.6
Not using family planning	0.10	0.026	0.0001*	0.028	0.0004*	1.9
Statistic	Value					
Joint Wald Statistic	120.01; *p* = 0.0000001					
Koenker (BP)Statistics	47.8; p = 0.0000001					
Breusch–Pagan statistic	75.5; *p* = 0.000001					

*=significant at alpha 5%

### Geographical weighted regression (GWR) model result

In a Geographically weighted regression model, the predictor variables of the GLR model (Anemia, home delivery, no (contraceptives use, not educated women proportion) were incorporated into a geographically weighted regression model. To determine the number of neighboring clusters for local regression, the bandwidth with the lowest AICc was chosen. The bi-square adaptive kernel function looks at an adaptive number of neighbors and the influence of these neighbors’ decays following a Gaussian distribution so that closer observations have the most weight. So local regression for adjacent clusters that have few data points will include clusters farther away. Comparing the global and the local model shows that the GWR model performs better than the GLR model.

**Fig. 6:**
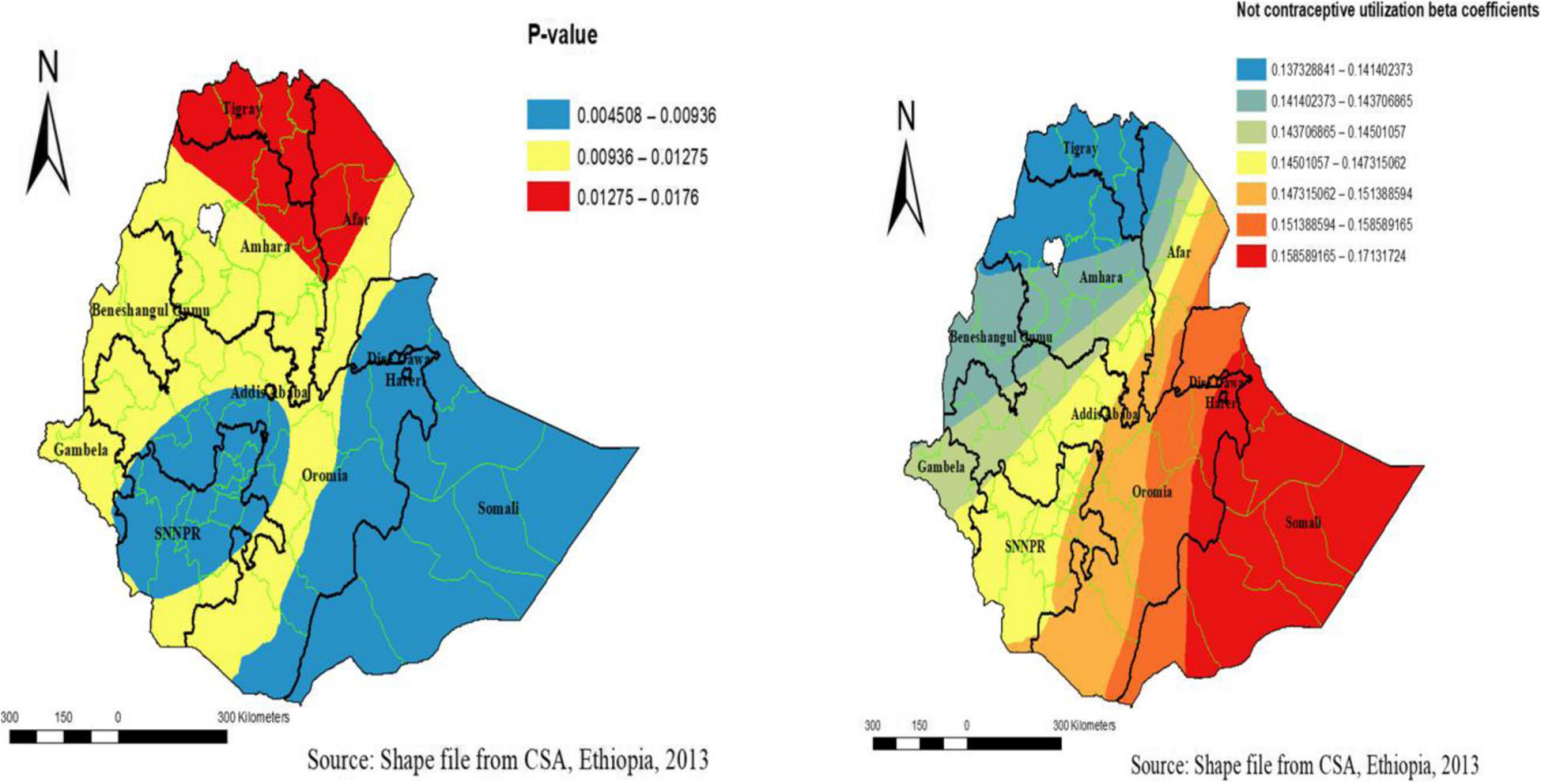
Geographically varying values of significance and coefficients per cluster for predictor variable not use contraceptive

No contraceptives use among women had different statistical significance in different parts of Ethiopia for HRFB among reproductive-age women. The coefficients of no contraceptives use vary spatially between 0.137 is Amhara and Region into 0.171 Somali, indicating that the effect of association different in different parts of Ethiopia. In the significant parts of Ethiopia, a 1% increase in no contraceptives use among women increases the prevalence of HRFB by 70%. No contraceptive use was significant across Ethiopia (Fig. 6).

**Fig. 7:**
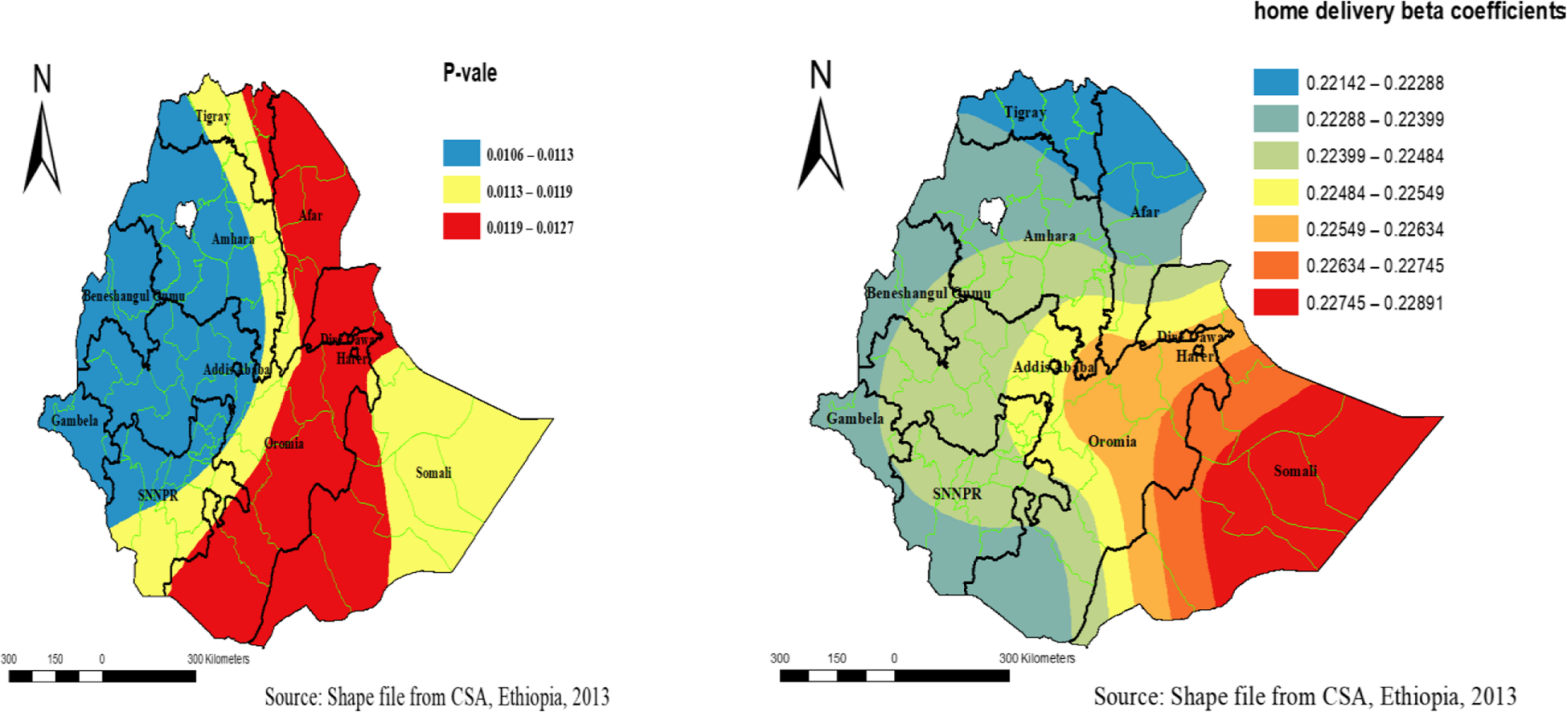
Geographically varying values of significance and coefficients of home delivery per cluster for high-risk fertility behavior in Ethiopia using Ethiopian Demographic and Health Surveys 2016

Home delivery among women had different statistical significance in different parts of Ethiopia for HRFB among reproductive-age women in Ethiopia. The geographically varied risk factors of home delivery range from 0.221 in Tigray to 0.228 in Somali (Fig. 7).

## Discussion

This study revealed that 76% of women had high-risk fertility behavior with a 95% confidence interval of 75.6 to 77.20%. This finding was lower than a study conducted in the Afar region of Ethiopia (86.3%) [25]. However, this finding was higher than the 2011 EDHS report 58% [3], 34% in Bangladesh DHS, 38.3% in Nepal, and 44.9% in India [26]. The possible explanation for the observed discrepancies might be that socio-demographic characteristics changes and increased fertility intention in society. Specifically, as compared with Asian countries such as Nepal, the socio-demographic characteristics are quite different and the health system variations could be the reason. Besides, in Ethiopia, child marriage is higher, which might be responsible for the increased magnitude of high risky fertility behavior [27].

This study revealed that the spatial distribution of HRFB was non-random in Ethiopia. Significant HRFB highly clustered at Somali and Afar. In line with this high proportion clustering, spatial scan statistics analysis revealed that 385 significant clusters were identified. A high HRFB observed in Somali and Afar, and a low proportion of HRFB observed in Amhara, Addis Ababa, Oromia, and SNNP. The observed geographical variation of HRFB across Ethiopia regions might be due to the regional variation health system infrastructure and this result is supported Ethiopian demographic survey report [3].

Geographically weighted regression has provided local parameter estimates of the model fit’s predictor variables vary spatially in Ethiopia. Home delivery and no contraceptives use were local level statistically significant predictor variables for HRFB among reproductive-age women in Ethiopia.

One of the obstacles to tackle maternal and child mortalities is High-risk fertility. This high-risk fertility is indirectly associated with home delivery because women who deliver at home with high-risk fertility had low service utilization of counseling about the benefit of optimal birth spacing [14].

Across regions of Ethiopia, the estimates of high-risk fertility behavior for women who deliver at home varied between 0.221 and 0.228. This variation in coefficients of high-risk fertility for those women who are delivering at home varied from region to region. Home delivery is a relatively stronger significant factor for high-risk fertility behavior in Amhara, Tigray, Afar, and Oromia regions than other regions. The statistically significant variation in estimates of high-risk fertility behavior across regions in Ethiopia might reflect the diverse socio-cultural setting differently responding to factors affecting fertility and child survival in the country than the perception of given community members to the issue in their contexts.

It should also be noted that there is a considerable variation in actual fertility level estimates across different regions in the country [3]. Therefore, the likelihood of getting exposed to high-risk fertility behavior is observed among areas experiencing high fertility and vice-versa, indicating that more children’s desire is a trigger of high-risk fertility [13].

Women who had no ever used contraceptive was associated with an increased occurrence of high-risk fertility behavior compared to those who had used. This finding is supported by other studies and evidence [13, 15] and DHS analytical study [28]. One of the purposes of contraceptive use is spacing birth and decreasing unintended pregnancies, which might affect the mother and child’s health. One of the basic postnatal intervention is family planning service provision for mothers to spacing birth intervals [18].

The study has some strengths. As Tobler’s first law of geography states that “Everything is related to everything else, but near things are more related than distant things” [29]. Based on Tobler’s first law of geography, HRFB was spatially autocorrelated. In the presence of spatial dependence and heterogeneity, the estimates obtained from the global model would be biased. Therefore, fitting the GWR model and knowing the spatial distribution of HRFB in Ethiopia regions provides important insight to policymakers and health planners and valuable hot spot maps used to more effective and cost-efficient nutrition intervention.

The study has also limitations: Since the data used in this study was cross-sectional data, which limits the conclusions about the causality of the factors on the dependent variable and Since 21 clusters did not have coordinated data we excluded in the analysis this may affect the estimated result.

## Conclusions

In Ethiopia, HRFB varies across regions. Statistically, a significant-high hot spot high-risk fertility behavior was identified at Somali and Afar. No contraceptive use and home delivery were statistically significant predictors (at a local level) in different regions of Ethiopia. Therefore, policymakers and health planners better to design an effective intervention program at Somali, and Afar to reduce high-risk fertility behavior and Special attention needs about health education on the advantage of contraceptive utilization and health facility delivery to reduce high-risk fertility behavior.

## Data Availability

The data was available from the corresponding author and we can provide upon request.

## Abbreviations

AICc: Corrected Akakies Information Criteria
ANC: Antenatal Care
EAS: Enumeration Areas
EDHS: Ethiopian Demographic and Health Survey
GWR: Geographically Weighted Regression
HRFB: High-Risk Fertility Behavior
MGWR: Multiscale Geographically Weighted Regression
SNNPR: South Nation Nationalities and People of Regions
